# Clinical analysis of conformal and intensity-modulated radiotherapy in patients with recurrent ovarian cancer

**DOI:** 10.1038/s41598-020-74356-7

**Published:** 2020-10-14

**Authors:** Hua Yang, Kaishuo Zhang, Zi Liu, Tao Wang, Fan Shi, Jin Su, Jintao Zhang, Juanyue Liu, Li Dai

**Affiliations:** 1 Department of Radiotherapy, Xijing Hospital of Air Force Military Medical University (the Fourth Military Medical University), Xi’an, Shaan’xi China; 2 Department of Radiotherapy, The First Affiliated Hospital of Xi’an Jiaotong University, Xi’an, Shaan’xi China; 3 Department of Radiotherapy, Xi’an Gao Xin Hospital, Xi’an, Shaan’xi China

**Keywords:** Gynaecological cancer, Ovarian cancer

## Abstract

We aimed to provide evidence for radiotherapy treatment regimens in patients with clinically recurrent ovarian cancer. We analyzed the survival and prognostic factors in 43 patients who were treated for recurrent ovarian cancer at 58 tumor sites using three-dimensional conformal radiotherapy (3D-CRT) or intensity-modulated radiotherapy (IMRT) during January 2006–December 2017. t years 1, 2, and 3, overall survival (OS) rate was 82.4%, 68.4%, and 57.9%; local control (LC) rate was 100%, 100% and 80%; recurrence free survival (RFS) rate was 86.8%, 66.6%, and 61.1%; and disease-free survival (DFS) rate was 79.7%, 56.7%, and 46.8%, respectively. The radiotherapy technique was determined to be an independent prognostic factor for survival; the survival rate of patients was significantly improved with IMRT compared to 3D-CRT (P = 0.035). Radiotherapy dose was an independent prognostic factor; survival rate improved when patients were treated with a radiation dose ≥ 60 Gy as compared to < 60 Gy (P = 0.046). Elective nodal prophylactic radiation therapy (ENRT) did not lead to a significant improvement in survival when compared to involved-field radiation therapy (IFRT). The toxicities of 3D-CRT and IMRT were tolerable. One patient (2.3%) had grade 3 acute gastrointestinal (GI) toxicity, 2 (4.6%) grade 3 late GI toxicity, 5 (11.6%) grade 3 hematological toxicity, and 2 (4.6%) had grade 4 hematological toxicity. IMRT improved LC and OS in patients with recurrent ovarian cancer after surgery and multiple chemotherapy; toxicities were tolerable. The IMRT technique and radiotherapy dose of ≥ 60 Gy had independent prognostic significance for the survival of such patients.

## Introduction

The mortality rate of ovarian cancer is the highest among all cancers of the female reproductive system. The primary pathological type is epithelial ovarian cancer. Surgery combined with chemotherapy, with or without targeted therapy, is the first choice for newly diagnosed patients. However, 70% of patients with advanced disease relapse even after achieving complete remission.

Chemotherapy is effective against advanced epithelial ovarian cancer, particularly with recent improvements to paclitaxel and carboplatin-based chemotherapy regimens^1^. However, multiple rounds of chemotherapy lead to low patient tolerance and chemotherapy resistance. The MITO-4 study has shown that in patients with ovarian cancer who received the carboplatin plus paclitaxel regimen, neurotoxicity persisted in 15%, 14%, and 11% of patients after 6 months, 1 year, and 2 years of chemotherapy, respectively^2^.

Ovarian cancer is moderately sensitive to radiotherapy^3^. Although radiotherapy is not the main treatment modality for ovarian cancer, it can be used as postoperative adjuvant therapy and palliative treatment for advanced and recurrent tumors. In particular, the development of three-dimensional conformal and intensity-modulated technologies has led to improvement in the local control rate of tumors^4^. Due to these techniques, the damage to the surrounding normal tissue can be minimized to a large extent, while the treatment dose on the tumor can be increased. Radiotherapy’s value is more evident in the treatment of patients with recurrent ovarian cancer. In recent studies, radiotherapy of patients with recurrent ovarian cancer increased 5-year survival rates when compared to literature values of less than 20%^4–10^.

There are currently no large-scale randomized studies on radiotherapy in the treatment of recurrent ovarian cancer. The most relevant research is mostly being performed at single-centers and using small-sample retrospective study design. Chunduryet al^4^reported that IMRT resulted in good local control, high treatment efficacy, and limited toxicities in recurrent chemo-refractory ovarian cancer. Nonetheless, current studies lack a standardized and reliable treatment design. In addition, most of these studies have multiple limitations: (1) The follow-up time is short, at approximately only 20 months. (2) The dose of local radiotherapy is relatively low, with a median dose of 50.4 Gy. (3) Comparison of treatment efficacy between IMRT and 3D-CRT in recurrent ovarian cancer is lacking. (4) Comparison between involved-field radiation therapy (IFRT) and elective nodal prophylactic radiation therapy (ENRT) is lacking.

Based on this background information, this study conducted a long-term follow-up and detailed clinical analysis of 43 patients with recurrent ovarian cancer who received 3D-CRT or IMRT. The patients’ characteristics and their survival-related factors were investigated. The results of this study could facilitate the development of individualized radiotherapy treatment plans for these types of patients and help avoid excessive or ineffective treatment.

## Materials and methods

### Baseline clinical characteristics

Between January 2006 and December 2017, 43 patients with recurrent epithelial ovarian cancer admitted to the Department of Radiotherapy of the First Affiliated Hospital of Xi’an Jiaotong University. All patients had provided written informed consent for the IOERT procedure. The patients were aged between 24 and 74 years, with a median age of 56 years. Patients had single or multiple metastases in the vaginal cuff, abdominal and pelvic cavities, or lymph nodes as assessed by physical examination and imaging testing (color Doppler ultrasound, CT, or MRI). All lesions were measurable. The patients’ general conditions、the sites and number of recurrent lesions were assessed. A total of 58 individual recurrent sites were treated with 3D-CRT or IMRT. The average dose of the group was 59.1 Gy (BED 45–82 Gy, α/β = 10). There are 19 patients received 3D-CRT and 24 patients received IMRT (There was no difference in baseline data between two groups. IMRT has not been widely used in pelvic metastases before 2011 in our center), 26 patients received IFRT and 17 patients received ENRT. Radiation sites included; the vaginal cuff in 14 patients, the pelvic cavity in 30 patients (including 3 patients with pelvic lymph nodes), the abdominal cavity in 12 patients (including 4 patients with abdominal lymph nodes), and 2 patients had supraclavicular lymph node metastasis. Baseline characteristics of patients are listed in Table 1.

**Table 1:** Baseline patient clinical characteristics.

Baseline characteristics	n (%)
**Age at RT in years (median; range)**	56 (24–74)
**Response to adjuvant chemotherapy**	
Sensitive	15 (34.9%)
Resistant	28 (65.1%)
**RT site**	
Vaginal cuff	14 (24.1%)
Pelvic cavity	25 (43.1%)
Abdominal cavity	8 (13.8%)
Lymph node(s)	11 (18.9%)
**Number of RT sites per tumor**	
Single	28 (65.1%)
Multiple (2 or 3)	15 (34.9%)
**Stage**	
I	1 (2.3%)
II	5 (11.6%)
III	27 (62.8%)
IV	10 (23.3%)
**Radiotherapy technology**	
3D-CRT	19 (44.2%)
IMRT	24 (55.8%)
**Range of radiotherapy**	
IFRT	26 (60.5%)
ENRT	17 (39.5%)
**Radiotherapy dose**	
≥ 60 Gy	28 (65.1%)
< 60 Gy	15 (34.9%)
**CA-125 (U/mL)**	
Before palliative RT	157.7
After palliative RT	114.3

RT, radiotherapy; 3D-CRT, three-dimensional conformal radiotherapy; IMRT, intensity-modulated radiotherapy; ENRT, Elective nodal prophylactic radiation therapy; IFRT, involved-field radiation therapy.

### Patients

In all cases, the potential benefits of treatment must be carefully weighed against the risks, particularly for patients who are referred after multiple operations and courses of chemotherapy. In our study, all patients underwent surgical resection of the primary tumor, bilateral salpingo-oophorectomy, total abdominal hysterectomy, and selective biopsies and resections of lymph nodes and omentum. Surgery was followed by adjuvant platinum-based chemotherapy for all patients, regimens included paclitaxel plus carboplatin (TC), paclitaxel plus cisplatin (TP), or docetaxel plus carboplatin (DC). The median time of local recurrence was 6 months after adjuvant chemotherapy; Recurrences before RT were treated with multiple second/third-line chemotherapy regimens and/or secondary cytoreduction. 11 patients underwent secondary cytoreduction for recurrent lesions.

Patients with poor chemotherapy efficacy, good physical condition and no more than three metastases were selected for radiotherapy. Patients were selected to receive ENRT when the number of recurrent lesions was 2–3 in pelvic. IFRT was used in patients with single recurrent lesion; three patients had positron emission tomography (PET) before RT.

### Radiotherapy

**Figure 1:**
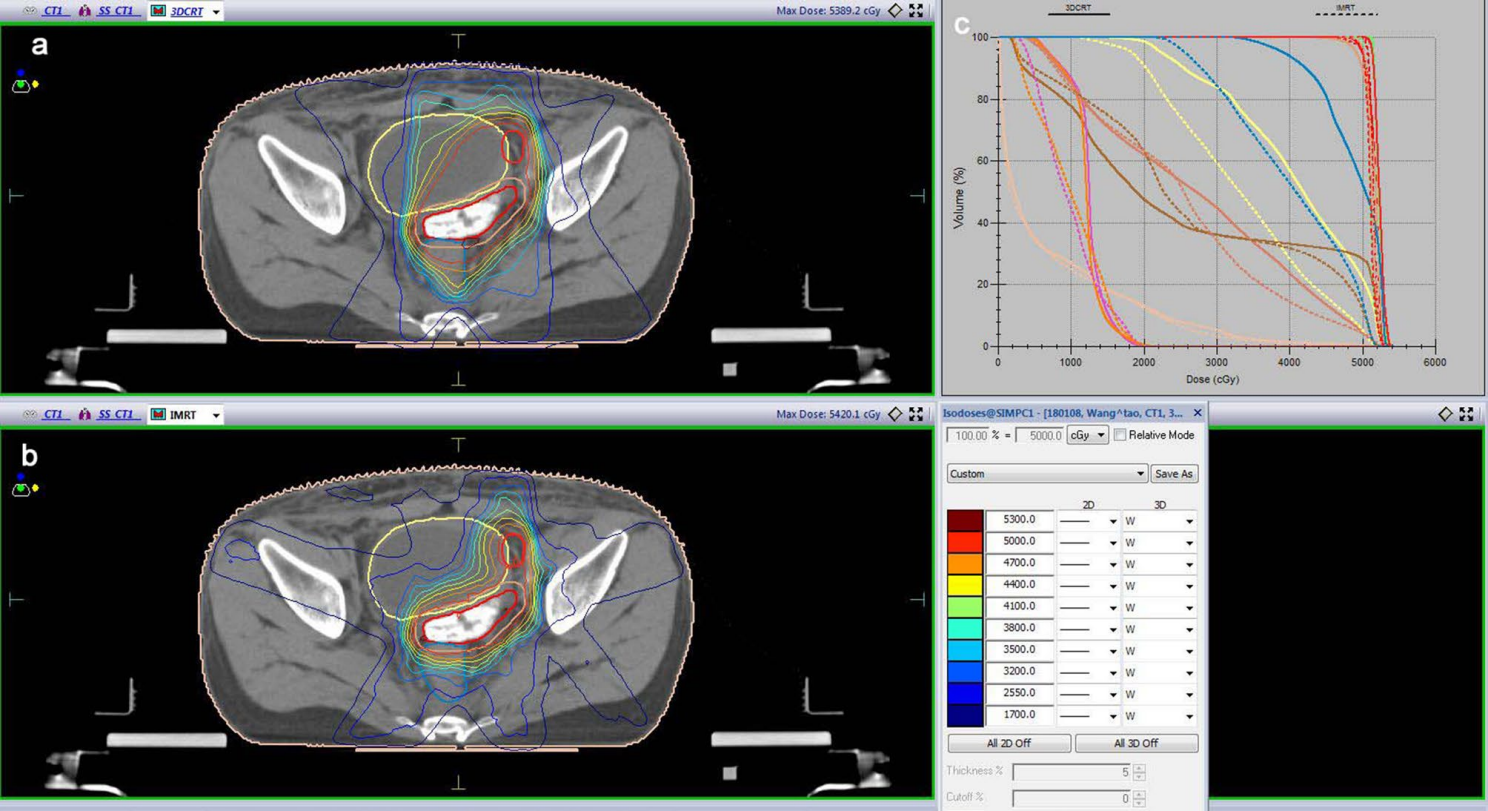
Dose distribution and DVH diagram between 3D-CRT and IMRT. (**a**) dose distribution of 3D-CRT; (**b**) dose distribution of IMRT; (**c**) Comparison of DVH diagram between 3D-CRT and IMRT.

Detailed procedure of IMRT: (1) positioning using CT simulation: patients were placed in the supine position with both arms wrapped around their head. A body mask was used to immobilize the patient to reduce positioning errors. A CT scanner was used to scan the abdominal and pelvic cavities. The scan was acquired from the upper abdomen to the pelvic floor using a 5 mm slice thickness; (2) Target volume delineation: the target volume was delineated at each CT slice, and the organs and target regions at risk were outlined. IFRT: The gross tumor volume (GTV) was determined at the site of the recurrent tumor or lymph node metastasis. The planning target volume (PTV) was determined by increasing GTV of 0.5–1.0 cm. The prescribed dose of PTV was 56-72 Gy, 1.8–2.2 Gy/fraction, 5 fractions /week. ENRT: The GTV was determined at the site of the recurrent tumor or lymph node metastasis. The clinical target value (CTV) encompassed the lymphatic drainage area in the GTV region. The planning GTV (PTV_boost_) was determined by increasing the GTV of 0.5 cm. The PTV was determined by increasing GTV of 0.5–1.0 cm. The prescribed dose of PTV was 45–50 Gy, 1.8–2.0 Gy/fraction. The prescribed dose of PTV_boost_ was 55-72 Gy, 2–2.2 Gy/fraction, 5 fractions/week. (3) Brachytherapy: 14 patients (32.6%) had vaginal recurrence, of which 4 had isolated metastatic nodules in the vagina or vaginal cuff. The vaginal cuff lesions were first irradiated externally using IMRT or 3D-CRT. After the initial dose reached 45–50 Gy, the appropriate brachytherapy applicator was selected according to local tumor size for intravaginal or intertissue implant irradiation after ENRT/IFRT. The average BED was up to 79.25 Gy; (4) Treatment planning: The IMRT technique during this study by using volumetric-modulated arc therapy (VMAT), The 95% dose prescribed covers 95% of the PTV (D95). Compared with normal three-dimensional conformal radiotherapy (3D-CRT), IMRT can provide better dose distribution and reduce the dose of organs at risk (OAR); IMRT plan need dose verification before the first treatment, and position verification is recommended once a week by image-guided radiotherapy (IGRT). 3DCRT plan do not need to do dose verification before treatment, and PA lateral view are verified every two weeks; the comparison of dose distribution between *3D-CRT* and *IMRT* is shown in Fig. 1. (5) Dose restriction to critical organs: The dose limit for the small intestine was V45 ≤ 200 cc, for the rectum it was V40 < 50%, and for the bladder it was V45 < 50%. The maximum dose for the spinal cord was 45 Gy or ≤ 50 Gy for 1 cc; for the lung it was V20 < 30%; and for the heart it was V30 < 20%. The acute and late toxicities were evaluated using the RTOG/EORTC classification criteria for radiation damage.

### Follow-up

All patients were followed-up regularly after receiving radiotherapy. The follow-up results were obtained during patient visits to the hospital for re-examination or by telephone. The Common Toxicity Criteria (CTC) 3.0 was used as the evaluation criteria for treatment-related adverse effects. The patients were followed-up every 3 months for the first year after treatment, every 6 months in years 2–3, and once every year from year 3 onwards. Outpatient follow-up examinations included a general physical examination, a contrast-enhanced CT of the lower abdomen, a pelvic MRI, a chest X-ray or chest CT, a B-scan ultrasound of the abdomen, and if necessary, a full-body bone scan or a cranial MRI.

### Statistical methods

Statistical analysis was performed using the SPSS 17.0 software. The Kaplan–Meier method was used to calculate the survival rates of patients, and the log-rank test was used to test their significance. The Cox proportional hazard model was used for multivariate analyses. P value of < 0.05 was considered statistically significant. Both overall survival (OS), local control (LC), recurrence free survival (RFS) and disease-free survival (DFS) were calculated from when the treatment started to when the event occurred.

### Ethical approval

All procedures performed in studies involving human participants were in accordance with the ethical standards of the institutional and/or national research committee and with the 1964 Helsinki declaration and its later amendments or comparable ethical standards. The present study was approved by the ethics committee of the First Affiliated Hospital of Xi’an Jiaotong University (No. XJTUIAF2018LSK-165).

## Results

### Follow-up

At the cutoff date of the last follow-up period on July 30, 2018, only 1 patient was lost to follow-up. The follow-up rate was 97.7%, with a median follow-up time of 46 (5.6–101.7) months.

### Efficacy

**Figure 2:**
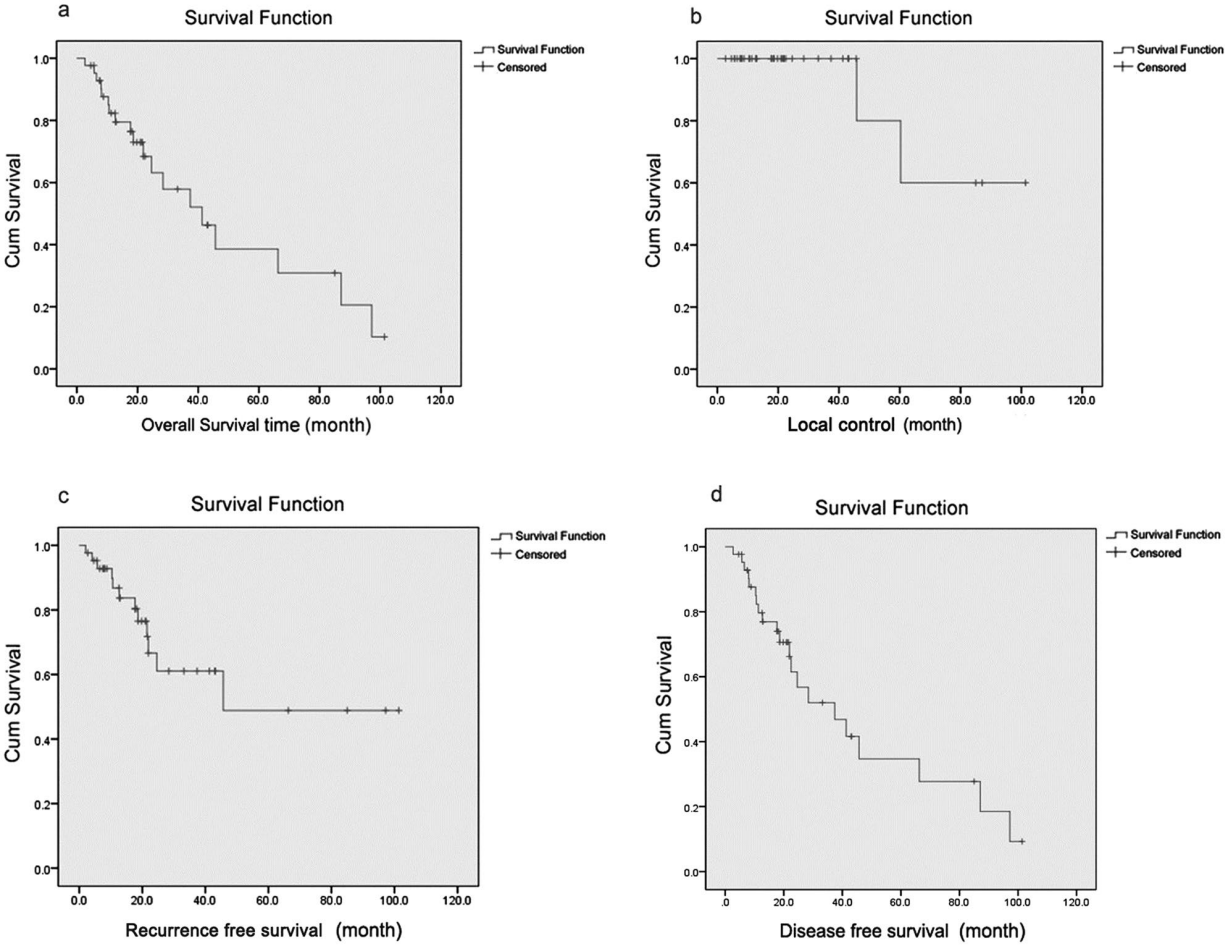
OS, LC, RFS and DFS of all patients. (**a**) OS of all patients. (**b**) LC of all patients. (**c**) RFS of all patients. (**d**) DFS of all patients.

In this study, a total of 43 patients and 58 individual recurrent sites were admitted, whose median age was 56 years (range 27–74 years). OS rate at years 1, 2, and 3 was 82.4%, 68.4%, and 57.9%; LC rate was 100%, 100%, and 80%;RFS rate was 86.8%, 66.6%, and 61.1%; and DFS rate was 79.7%, 56.7%, and 46.8%, respectively (Fig. 2).After completion of treatment, 14 of 43 patients relapsed. In-field recurrence occurred in 2 patients, out-of-field recurrence occurred in 12, and the median recurrence time was 18.6 months (2–66.3 months).In this study, 11 patients had lymph node metastasis.

### Prognostic factors

**Figure 3:**
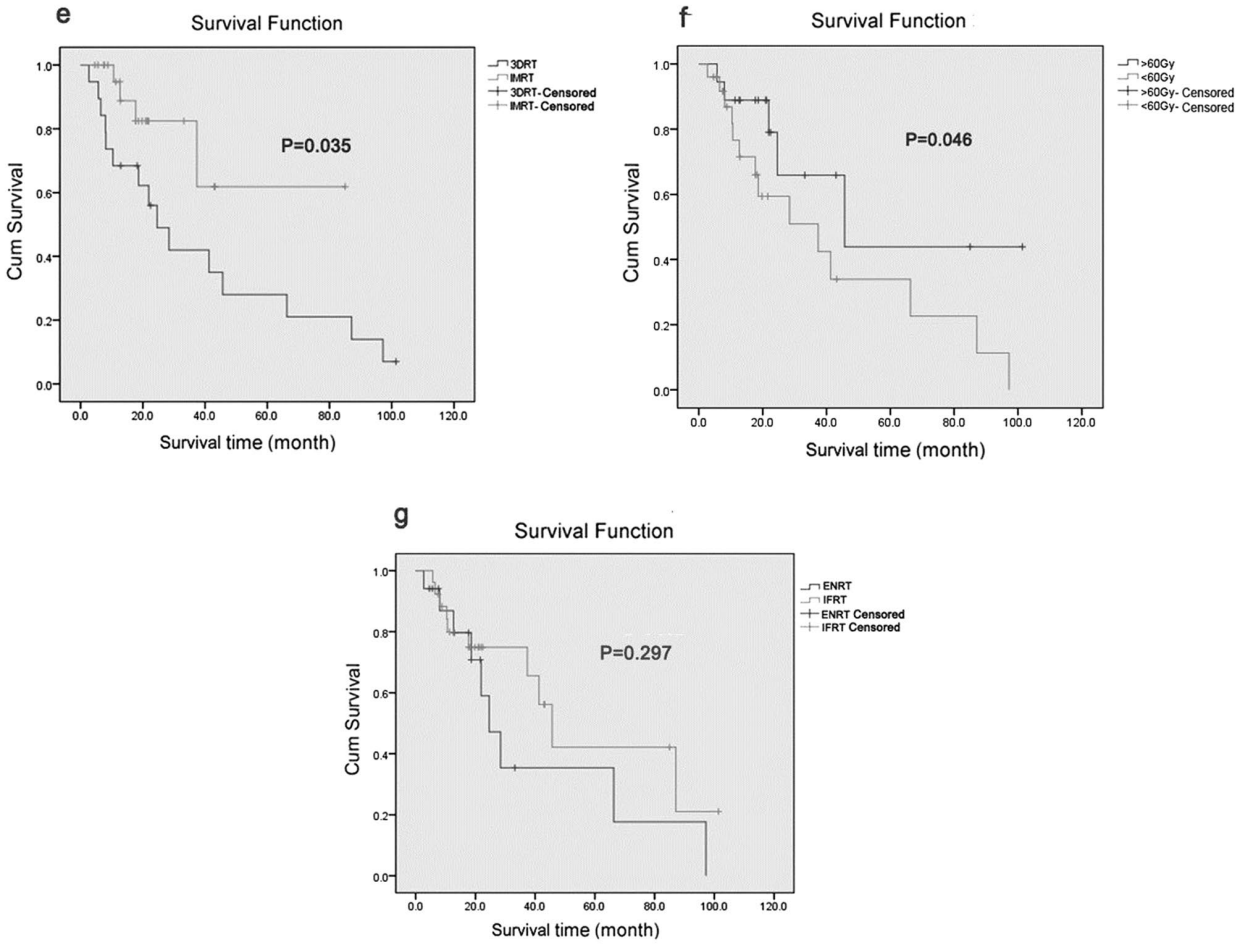
OS of patients in different groups. (**e**) OS comparison of patients between 3D-CRT and IMRT. (P = 0.035). (**f**) OS comparison of patients between ≥ 60 Gy and < 60 Gy. (P = 0.046). (**g**) OS comparison of patients between ENRT and IFRT (P = 0.297).

Cox regression analysis showed that the radiotherapy technique and radiotherapy dose were independent prognostic factors for the survival of patients with recurrent metastatic ovarian cancer (Table 2). IMRT significantly improved patient survival compared to 3D-CRT (P = 0.035).A radiation dose of ≥ 60 Gy significantly improved patient survival compared to a dose of < 60 Gy (P = 0.046).ENRT did not lead to a significant improvement in survival when compared to IFRT (P = 0.297) (Fig. 3).

**Table 2:** The results of the multivariate analysis.

Characteristics	β	SE	Wald	df	Sig	Exp(β)
Radiotherapy technology	0.257	0.046	10.54	1	0.035	1.346
Range of radiotherapy	0.824	0.094	3.68	1	0.297	2.089
Radiotherapy dose	0.544	0.056	9.86	1	0.046	0.628

### Toxicities

Patients tolerated 3D-CRT and IMRT well, and the toxicities were acceptable. Grade 3/4 toxicities occurred in patients who received combined concurrent chemotherapy. One patient(2.3%) had grade 3 acute gastrointestinal (GI) toxicity, 2 (4.6%) grade 3 late GI toxicity, 5 (11.6%)grade 3 hematological toxicity, and 2 (4.6%) had grade 4 hematological toxicity. No grade 5 acute or late toxicities were observed (Table 3).

**Table 3:** Grade 3 and 4 toxicities.

Grade	Acute toxicities		Late toxicities	
	Hematological	Gastrointestinal	Hematological	Gastrointestinal
Grade 3	5 (11.6%)	1 (2.3%)	0	2 (4.6%)
Grade 4	2 (4.6%)	0	0	0

## Discussion

The treatment of recurrent ovarian cancer remains a challenging issue. Even with the rapid development of chemotherapeutic agents, significant survival benefit has yet to be achieved. Furthermore, patients have poor tolerance and a reduced quality of life after undergoing multiple courses of chemotherapy. With the advancement of radiotherapy technology and the corresponding reduction of toxicities, radiotherapy has been widely utilized in the treatment of recurrent ovarian cancer. Studies on various radiotherapy techniques, including salvage radiotherapy for locally recurrent sites, intracavitary radiotherapy, and prophylactic pelvic radiotherapy have achieved satisfactory results^4,9–13^. Definitive IFRT can yield excellent local control, protracted disease-free intervals, so RT was considered a tool in the curative management of locoregionally-recurrent ovarian cancer^8^. However, these studies have certain limitations: (1) The follow-up time is relatively short, mostly at approximately 20 months; (2) The dose of local radiotherapy is relatively low, with a median dose of 50.4 Gy; (3) The comparison of treatment efficacy between IMRT and 3D-CRT in recurrent ovarian cancer is lacking; (4) The comparison between IFRT and ENRT is lacking. In this study, 43 patients with recurrent ovarian cancer treated with 3D-CRT or IMRT were followed up for a long time and analyzed in detail.

Radiotherapy can significantly improve LC of recurrent ovarian cancer; the treatment efficacy was very satisfactory. However, these patients are prone to recurrence in other parts of the pelvic cavity, leading to the reduction of RFS. In our study, the 2-year OS, LC, RFS rates were 68.4%, 100%, and 66.6%, respectively, which were all higher than those reported by Chundury et al. (63%, 82%, and 11%, respectively). This might be due to the higher radiation dose used in the present study, which had an average dose of 59.1 Gy (BED 45–82 Gy)^4,9,10^. Previous studies have shown that using 50 Gy as the BED limit increased the incidence of complete remission (CR).However, when 60 Gy was used as the BED limit, the CR rate showed no significant improvement^4,9,10^. In contrast, the present study showed that a radiotherapy dose of ≥ 60 Gy significantly improved the survival of patients (P = 0.046, Fig. 3). This discrepancy might be due to the use of conventional radiotherapy techniques in previous studies, where an increase in radiation dose could cause more damage to normal tissues and negatively affect survival. However, our study showed that IMRT could improve localization, prolong survival, and reduce normal tissue side effects.

Lymph nodes are a common metastatic site for recurrent ovarian cancer. Some studies have indicated that the most common sites of metastatic recurrence are para-aortic lymph nodes and supraclavicular lymph nodes. Current radiotherapy for recurrent lymph nodes mainly targets the involved region, which includes the entire anatomic region of the metastatic lymph nodes^9,10^. The IMRT technique displays an excellent dose advantage for irradiation of the recurrent lymph node region. The dose can be adjusted to better target the tumor site while simultaneously reducing the dose to the surrounding normal tissues. This protects the normal tissues and organs around the enlarged lymph nodes. This study also showed that IMRT significantly improved patient survival and conferred better tolerability in patients when compared to 3D-CRT. 11 patients had lymph node metastasis in this study. Two had supraclavicular lymph node metastasis. Five had pelvic lymph node metastasis, and four had para-aortic lymph node metastasis. All patients with lymph node metastases were treated with IMRT of 45–50.4 Gy/25–28 fraction at the lymphatic drainage area, and with a simultaneouslyincreased dose of 55–61.6 Gy/25–28 fraction at the metastatic lymph nodes. All of these lymph node metastases disappeared completely. The treatment efficacy was satisfactory.

In this study, 14 patients (32.6%) had vaginal recurrence, of which 4 had isolated metastatic nodules in the vagina or vaginal cuff. The vaginal cuff lesions were first irradiated externally using IMRT or 3D-CRT. After the initial dose reached 45–50 Gy, the appropriate brachytherapy applicator was selected according to local tumor size for intravaginal or intertissue implant irradiation. The average BED was up to 79.25 Gy, and the 2-year local control rate was 100%. However, in most patients, the small intestine descends to the lower part of the pelvic cavity and occasionally adheres to the vaginal cuff. Therefore, attention should be paid to the distance between the vaginal cuff, the lesion, and the small intestine during radiotherapy when selecting the radiation dose. This can prevent excessive dosing of the small intestine that may lead to stenosis or perforation^7,14^.

Dissemination to the abdomen and pelvis is the most common type of metastasis in ovarian cancer. Additionally, multiple lesions within the abdominal and pelvic cavities often accompany ovarian cancer recurrence. The goal of prophylactic whole pelvic radiotherapy is to reduce the chance of pelvic occurrence by lowering the risk of peritoneal implantation and destroying subclinical lesions. The standard dose for pelvic cavity radiotherapy is 45–50 Gy (1.8 Gy/fraction). A 2011 retrospective analysis compared pelvic cavity radiotherapy plus chemotherapy versus chemotherapy alone in patients with recurrent ovarian cancer. Patients who received pelvic cavity radiotherapy plus chemotherapy had a higher PFS compared to patients treated with chemotherapy alone (56% vs 36%, P = 0.032)^15^. In this study, 17 patients received hypofractionated IFRT with prophylactic pelvic cavity radiotherapy (i.e., ENRT) and 26 received IFRT only. The results showed that ENRT did not significantly improve the survival rate when compared to IFRT (P = 0.297) (Fig. 3).This may reflect the fact that the majority of patients in the ENRT group were at high risk.

There were limitations to this study. Despite, long-term and detailed follow-ups, the initial sample size were small. Thus, a larger sample size and additional research data are needed to further support these conclusions. Therefore, this factor was not included in the prognosis analysis of the patients.

In conclusion, the clinical treatment of recurrent ovarian cancer is a challenge. Although treatment methods have improved, the overall prognosis of patients remains poor. Systemic chemotherapy using paclitaxel plus platinum-based agents is still the first-line treatment for this disease. The results showed that IMRT could improve the LC and OS of patients with recurrent ovarian cancer after operation and multiple chemotherapy. It may provide a meaningful treatment for patients with refractory recurrent metastatic ovarian cancer. The observed toxicities were acceptable. Survival rate of patient was significantly improved with IMRT as compared to 3D-CRT, and with a radiation dose of ≥ 60 Gy as compared to doses < 60 Gy. ENRT did not lead to a significant improvement in survival when compared to IFRT. We expect that the results of this study will help clinicians better evaluate the prognosis of patients with recurrent ovarian cancer and serve as a guide for the selection of treatment plans.
